# Therapeutic and immunomodulatory potentials of mesenchymal stromal/stem cells and immune checkpoints related molecules

**DOI:** 10.1186/s40364-024-00580-2

**Published:** 2024-03-21

**Authors:** Ali Hazrati, Kosar Malekpour, Hossein Khorramdelazad, Samira Rajaei, Seyed Mahmoud Hashemi

**Affiliations:** 1 Department of Immunology, School of Medicine, Tehran University of Medical Sciences, Tehran, Iran; 2 Department of Immunology, School of Medicine, Iran University of Medical Sciences, Tehran, Iran; 3 Department of Immunology, Faculty of Medicine, Rafsanjan University of Medical Sciences, Rafsanjan, Iran; 4 Department of Immunology, School of Medicine, Shahid Beheshti University of Medical Sciences, Tehran, Iran

**Keywords:** Immune checkpoints, Mesenchymal stromal/stem cell, Immunomodulation, Extracellular vesicles, Cancer

## Abstract

Mesenchymal stromal/stem cells (MSCs) are used in many studies due to their therapeutic potential, including their differentiative ability and immunomodulatory properties. These cells perform their therapeutic functions by using various mechanisms, such as the production of anti-inflammatory cytokines, growth factors, direct cell-to-cell contact, extracellular vesicles (EVs) production, and mitochondrial transfer. However, mechanisms related to immune checkpoints (ICPs) and their effect on the immunomodulatory ability of MSCs are less discussed. The main function of ICPs is to prevent the initiation of unwanted responses and to regulate the immune system responses to maintain the homeostasis of these responses. ICPs are produced by various types of immune system regulatory cells, and defects in their expression and function may be associated with excessive responses that can ultimately lead to autoimmunity. Also, by expressing different types of ICPs and their ligands (ICPLs), tumor cells prevent the formation and durability of immune responses, which leads to tumors' immune escape. ICPs and ICPLs can be produced by MSCs and affect immune cell responses both through their secretion into the microenvironment or direct cell-to-cell interaction. Pre-treatment of MSCs in inflammatory conditions leads to an increase in their therapeutic potential. In addition to the effect that inflammatory environments have on the production of anti-inflammatory cytokines by MSCs, they can increase the expression of various types of ICPLs. In this review, we discuss different types of ICPLs and ICPs expressed by MSCs and their effect on their immunomodulatory and therapeutic potential.

## Introduction

MSCs are a group of multipotent stem cells usually found in all body tissues [1]. These cells can usually be isolated and expanded in the laboratory from bone marrow, adipose tissue, umbilical cord, etc. [2]. According to past studies, it has been shown that these cells can be used in the treatment of various types of diseases, such as autoimmune and infectious, as well as in treatments based on tissue regeneration due to their multiple characteristics [3, 4]. It has also been shown that the use of MSCs in live, apoptotic, and dead states can have different therapeutic effects [5, 6]. One of the features that has led to a lot of attention to these cells is their ability to self-renew and differentiate [7, 8]. MSCs in therapeutic applications, after being injected into animal models or patients, maintain their self-renewal ability for an acceptable period, proliferate and differentiate in the damaged tissue-related cells [9], and help restore the damaged tissue [10, 11]. These cells produce various growth factors, including VEGF, PDGF, EGF, HGF, IGF-1, FGF-4, FGF-2, FGF-7, BMP-7, and FGF-9 [12, 13], which leads to the growth, proliferation, and differentiation of healthy cells in the tissues also increase and prevent the destruction of other tissue cells [14, 15]. It is also known that MSCs have a high immunomodulatory potential [16–18]. Investigations to find the reasons for this kind of therapeutic effect of MSCs have shown that MSCs perform this action by producing anti-inflammatory cytokines, including IL-13, IL-16, IL-10, and TGF-β [19]. In addition, various chemokines are produced by MSCs, which can increase the recruitment of immune system regulatory cells to the injury site [20, 21]. In addition to the mentioned mechanisms, studies have shown that MSCs can perform their therapeutic functions by producing extracellular vesicles (EVs), especially exosomes (EXOs) [22–24]. EXOs are secreted from the cell after formation in the multivesicular body (MVB) [25]. These vesicles contain various substances, including proteins (enzymes, cytokines, growth factors, chemokines), nucleic acids (single-stranded and double-stranded DNA, mRNA, miRNA, lncRNA, and circRNA), and lipids [26–28]. Also, novel studies have shown that exosomes can carry whole mitochondria or mitochondria-related subunits to the damaged cells and restore their functions [29]. However, many studies state that changes in the culture conditions of MSCs can increase their therapeutic ability [2, 30, 31]. For example, 3D culture of MSCs can increase their immunomodulatory capacity by increasing cytokine and exosome production [32]. Also, placing them in conditions such as hypoxia [33], cultivation in inflammatory conditions [34], and using genetic engineering methods [35] can increase these cells' theraputic efficacy. But how this modifications (culture in inflammatory condition) takes place was questioned and debated for years. Some studies have highlighted the importance of exosomes and EVs derived from MSCs in their therapeutic potential [36]. However, some other studies have shown that the therapeutic effects of MSCs occur through cell-to-cell interaction [37–40] (Table 1).

**Table 1:** MSCs produced immunomodulatory mediators

No	Immunomodulatory Mediators	Examples	Functions	Ref
1	Extracellular vehicles	Exosomes	1. Carry cytokines, growth factors, chemokines, and other component of cells 2. Effect on target cell proliferation, immune response, oxidative stress, etc 3. ↓ Liver and lung fibrosis	[41–45]
		Microvesicles		
		Apoptotic bodies		
2	Chemokines and receptors	CCL2, CCR2, CCL3/4, CCR5, CCL5, CCR1, CCL20, CCR6, CCL21, CCR7, CXCL1, CXCR2, CXCL1/2/8, CXCR1, CXCL1/5, CXCR2, CXCL8, CXCL8, CXCL12, CXCR4, CXCL16, CXCR6, CX3CL1	1. MSCs homing 2. ↑ Lymphocyte recruitment and MSC differentiation 3. Tumor metastasis 4. ↑ Macrophage polarization and tumor progression 5. Neuroprotective phenotype of microglia 6. ↑ CD4 ^+ ^T cell migration	[13, 46–50]
3	Growth factors	VEGF, PDGF, EGF, HGF, IGF-1, FGF-4, FGF-2, FGF-7, BMP-7, and FGF-9	1. Pro-and anti-angiogenesis effects 2. ↑ Tissue remodeling 3. ↑ Tissue regeneration 4. ↓ Death of damaged cells	[12, 51–54]
4	Inflammatory cytokines	IL-6	↑ Maturation and support of the survival of human antibody-secreting cells	[55–58]
		IL-7	1. ↑ The proliferation of colitogenic CD4^+^ memory T cells 2. B cell development	
		IL-8 and migration inhibitory factor (MIF)	↑ The function and survival of neutrophils	
		IL-28	↑ Cancer cells apoptosis	
5	Anti-inflammatory cytokines	LIF	Inhibiting T-cell proliferation	[59–63]
		IL-10	Induce MSCs the secretion of HLA-G5 soluble isoform and suppress innate immunity	
		IL-4	Polarize microglia to the anti-inflammatory phenotype	
		TGF-β	1. ↓ The content of Th2 cytokines (IL-4, IL-5, and IL-13) in bronchial lavage 2. ↓ Th2 type immunoglobulins in serum 3. Induces regulatory T cells 4. ↓ M1 macrophage phenotype induction	
6	ECM components	Collagens, vimentin, integrin, galectins,	1. Support the formation and stabilization of vessels	[9, 64, 65]
7	Enzymes	Inducible nitric oxide synthase (iNOS)	1. Metabolizes the essential amino acid, tryptophan, into downstream kynurenines 2. Promoting immune tolerance 3. Keep pro-inflammatory signaling in check	[66, 67]
		Indoleamine 2,3-dioxygenase (IDO)	Controlling M1 macrophage differentiation	[68]
		Ectoenzymes such as CD73 and CD39	1. Suppresses immune cells functions 2. ↑ Tumor cell survival and metastasis	[69, 70]
8	Mitochondria	Whole mitochondria or its subunits	1. ↑ ATP production 2. ↑ Oxidative phosphorylation 3. ↑ Expression of genes involved in lipid metabolism and glycolysis 4. ↑ Treg differentiation and proliferation 5. ↓Conventional T cell activation and proliferation 6. ↑ Anti-inflammatory cytokines production 7. ↑ Macrophage M2 polarization	[29, 71, 72]

Molecules belonging to a family called ICPs play an important role in regulating immune system responses induced by regulatory cells, including Tregs [73, 74]. Therefore, in recent years, researchers have investigated whether these molecules and their ligands are expressed on the surface of MSCs and their role in regulating immunity induced by these cells. The results show that MSCs express different types of ICPs and their ligands, and they can be called quasi-immune regulatory cells. Studies show that the functions of ICPs and their ligands play a role in the therapeutic ability of MSCs in the treatment of inflammatory diseases such as autoimmune. In this review, we collect published information about the expression of ICPs and their ligands (ICPLs) on the MSCs' surface. First, we will talk about ICPs, their functions, and their role in disease and treatment, and then we will talk in detail about the studies on each of the ICPs expressed by MSCs.

## Immune checkpoints (ICPs) in disease and treatment

ICPs are usually considered as membrane receptors present on immune cells, while the ICPLs are expressed on the so-called “target cells” (namely, tumor cells, APCs, and stromal cells of different origins). This notion was modified markedly with the finding that CTLA-4, the major immune checkpoint molecule expressed on CTLs, can be expressed not only in lymphocytes but also by tumor cells [75, 76] and DCs [77]. As mentioned, the primary function of ICPs is the immune system response homeostasis (especially T cell responses) and preventing inappropriate responses [78]. ICPs play an essential role in peripheral tolerance and prevent the development of autoimmunity [79–81]. At the same time, tumor cells can express ICPs and prevent immune responses against these cells during tumor growth and development [82, 83]. Table 2 briefly shows the different types of ICPs, their expressing cells, and their roles (Table 2).

**Table 2:** Summary of molecules involved in the immune regulation, their ligands, and functions

No	Checkpoint receptor	Expressed cells examples	Ligand	Affected cells examples	Functions	Ref
1	CTLA-4 (CD152)	1. Regulatory T cells 2. Activated T cells 3. Monocyte-derived dendritic cells 4. B cells 5. NK cells 6. Tumor cells	1. CD80 2. CD86	1. Dendritic cells 2. Macrophage 3. Monocytes	1. Mediate opposing functions in T cell activation 2. Inhibits T-cell activation and proliferation 3. ↓ Cytotoxic T lymphocytes 4. Hemoestasis of immune responses 5. Play a crucial regulator role in self-tolerance	[77, 84–86]
2	PD-1 (CD279)	1. Activated T cells 2. NK cells 3. B cells 4. Macrophages 5. Several subsets of DCs 6. Innate-like lymphocytes (ILCs)	1. PD-L1 2. PD-L2 3. Galectin 9	1. Cancer cells 2. PD-L1 expressed by different cells 3. PD-L2 expression is restricted to DC and macrophages	1. Inhibit the effector function of CD8^+^ T 2. ↓ T cell proliferation 3. ↓ T cell cytokine production 4. Affect on epigenetic programs in tumor-infiltrated CD8^+^ T 5. ↑ T cell exhaustion	[87–90]
3	TIM-3 (CD366)	1. IFN-γ-producing T cells 2. Terminally exhausted T cells 3. FoxP3^+^ Treg cells 4. Macrophages 5. DCs 6. Tumor cells 7. NK/NKT cells	1. Galectin 9 2. Ceacam1 3. HMGB1 4. phosphatidylserine	1. Th1 cells 2. CD8^+^ T cell	1. ↑ Aggregation and death of Th1 cell in vitro 2. Inhibiting Th1 responses and the production of TNF-α and IFN-γ 3. Role in dominating the tumor-infiltrating CD8^+^ T cell pool in some cancer types	[91–94]
4	LAG-3 (CD223)	1. Regulatory T cells 2. Activated CD4^+^ T cell 3. Activated CD8^+^ T cell 4. Exhausted CD8 + T cells 5. NK cells 6. MAIT 7. γδT cells 8. iNKT cells 9. Tr1	1. MHC class II 2. FGL-1 3. α-synuclein fibrils 4. Gal-3 5. LSECtin	1. Antigen-presenting cells 2. Somatic cells	1. Improves the function of Treg cell 2. ↓ T cells proliferation 3. ↓ T cell cytokine and granzyme production 4. Suppress tumor-specific T-cell functions	[95–100]
5	TIGIT and CD96	1. Intratumoral T cells 2. Regulatory T cells 3. Follicular T helper cells 4. NK cells 5. Tumor cells	1. CD155 (PVR) 2. CD112 (PVRL2, nectin-2) 3. Fap2 4. Nectin-4	1. DCs 2. Tumor cells	1. Act as a negative regulator of T cell functions 2. ↓ TCR expression and signaling 3. Upregulates CCR8 expression in Treg, which may promote migration to tumor tissue 4. Suppress Th1 and Th17 cells function 5. ↑ immunosuppressive cytokine production like IL-10	[101–105]
6	BTLA (CD272)	1. Activated T cells 2. Naïve T cells 3. Naïve B cells 4. NK cells 5. Macrophages 6. DCs	HVEM	1. T cells 2. B cells 3. NK cells 4. DCs 5. Myeloid cells 6. Tumor cells	1. Blocks B and T cell activation 2. ↓ B and T cell proliferation 3. ↓ B cell, T cell, and DC cytokine production 4. ↑ Treg and Th2 differentiation	[106–110]
7	A2AR	1. Regulatory T cells 2. Cytotoxic T cells 3. Macrophages 6. Tumor cells	Adenosine	Adenosine is produced by CD39 and CD73 expressing cells and released to the extracellular environment	1. Modulate alpha-synuclein aggregation and toxicity 2. ↑ IL-10 production by immune cells 3. ↓ NF-κB signaling pathway 4. ↓ Immune cells protein synthesis 5. ↓ Immune cell proliferation and survival	[111, 112]
8	CD24	1. Regulatory T cells 2. B10 regulatory B cells 3. Hematopoietic cells 4. Neutrophils 5. Eosinophils 6. Macrophages 7. Dendritic cells 8. Non-hematopoietic cells 9. Tumor cells	1. Siglec-10 2. P-selectin	1. B cells 2. Monocytes 3. Dendritic cells 4. Activated CD4^+^ T cells	1. T-cell homeostatic proliferation in lymphopenic hosts 2. Promoting cell migration 3. Role in the development of the human central nervous system 4. ↑ Tumor cell proliferation	[113–116]
9	SIRPα (CD172a)	1. Monocytes 2. Granulocytes 3. DCs 4. Hematopoietic stem cells	CD47	1. Thymocytes 2. T and B cells 3. Monocytes 4. Platelets 5. Erythrocytes 6. Neural cells 7. Fibroblasts 8. Tumor cell	1. Act as a negative regulator of the PI3K and MAPK 2. Leads to reduced responsiveness to tyrosine kinase ligands 3. ↑ Cancer cell proliferation 4. ↓ T cell-mediated antitumor responses 5. Prevents the phagocytic synapse formation on myeloid cells 6. Prevent virus-infected and bacteria-infected cells immune-mediated elimination	[117–119]
10	LILRB1	1. B cells 2. T cells 3. Monocytes and macrophages 4. Dendritic cells 5. NK cells 6. Basophils 7. Eosinophils 8. γδ T cells	1. HLA class I molecules including HLA-A, HLA-B, HLA-C, HLA-E, HLA-F, and HLA-G 2. S100A8 3. S100A9 4. RIFINs	1. Somatic cells 2. APCs	1. Inhibits super-antigen–dependent cell cytotoxicity 2. Impairs B-cell antibody production and proliferation 3. Role in immune tolerance 4. Alters DCs differentiation 5. Chang DCs capacity in cytokines production	[120–125]
11	VISTA	1. Myeloid lineage-related cells, including MDSCs 2. Haematopoietic cells 3. Tumor cells 4. Activated CD4^+^ T cells 5. Activated CD8^+^ T cells	1. PSGL-1 2. VSIG3	1. Brain cells 2. Testis cells 3. Skeletal muscle cells 4. Tumor cells	1. Suppresses proliferation of T cells 2. Bluntes T cell cytokines production and activation markers 3. Induces Foxp3 expression in T cells 4. ↓ Tumour-infiltrating CD8 + T cells in vivo 5. ↓ IFNγ, IL-2, IL-6 and IL-12 production from T cells 6. ↓ Toll-like receptor (TLR) signaling 7. ↓ Cell migration 8. ↑ IL-10 and other anti-inflammatory mediators production	[126–131]
12	Killer cell immunoglobulin-like receptors (KIR)	1. NK cells 2. T cells	1. MHC class I	Nearly every normal nucleated cell	1. Development of NK cells 2. Tolerance of NK cells 3. Activation of NK cells 4. ↓ T cell effector function 5. ↓ T cell activation-induced cell death (AICD)	[132–134]
13	C-lectin-type-inhibitory receptors (CLIRs), including DCIR	1. Myeloid cells, including DCs and macrophages 2. Osteoclasts	Asialo-biantennary N-glycan (NA2)	1. Bone cells 2. Myeloid cells	1. ↓ TLR8-dependent IL-12 and TNFα production from DCs 2. ↓ Pro-inflammatory cytokines IL-1β and IL-6 frpm macrophages 3. ↓ IL12p70 production and DC-dependent TH1 skewing 4. Inhibited osteoclastogenesis	[135, 136]
14	Inhibitory Siglec receptors	1. Immune effector cells 2. Trophoblasts 3. Myelin-forming cells 4. Stromal cells	Sialoglycans such as α2,3/6-linked sialic acid	1. Stromal cells 2. Immune cells 3. Cancer-associated fibroblasts	1. ↑ Production of immunosuppressive cytokines 2. Dampen activation of antigen-presenting cells 3. Inhibit NK activation 4. ↑ Differentiation of MQs immunosuppressive M2 phenotype 5. ↑ Antigen-specific Tregs population 6. ↓ Proliferation of effector T cells 7. Shaping MSC/CAF immunosuppression in the TME	[137–139]

*APCs* Antigen-presenting cells, *Gal-3* Galectin-3, *LSECtin* Lymph node sinusoidal endothelial cell C-type lectin, *MAIT* Mucosal-associated invariant T cells, *iNKT* Invariant natural killer T cells, *Tr1* CD4^+^ type 1 T regulatory, *HVEM* Herpesvirus entry mediator, *PI3K* Phosphatidylinositol 3-kinase, *MAPK* Mitogen-activated protein kinase, *RIFINs* Repetitive interspersed families of polypeptides, *VSIG3* V-Set and Immunoglobulin domain containing 3, *PSGL-1* P-selectin glycoprotein ligand 1, *VISTA* V-domain Ig suppressor of T cell activation, *MDSCs* Myeloid-derived suppressor cells, *LILRB1* Leukocyte Immunoglobulin Like Receptor B1, *SIRPα* Signal regulatory protein α, *A2AR* Adenosine A2A receptor, *BTLA* B and T lymphocyte attenuator, *TIGIT* T cell immunoreceptor with Ig and ITIM domains, *LAG-3* Lymphocyte activation gene 3, *TIM3* T cell immunoglobulin and mucin domain-containing protein 3, *PD-1* Programmed cell death protein 1, *PD-L* Programmed death-ligand, *CTLA-4* Cytotoxic T-lymphocyte-associated antigen-4

The expression of ICPs by tumor cells and vesicles produced by them (exosomes and MVs) lead to the differentiation of T cells to Treg [140, 141], the differentiation of macrophages to the M2 phenotype [142, 143], the reduction of the cytotoxic T lymphocytes (CTLs) functions [144, 145], the reduction of the immune cells recruitment to the tumor site, the increase of the recruitment of myeloid-derived suppressor cells (MDSCs) and Tregs [146], and induces of exhaustion [147], senescence and apoptosis in immune cells [140, 148]. It has also been shown that ICPs play an important role in the pathogenesis and persistence of infections related to malaria [149], human immunodeficiency virus (HIV) [150], and hepatitis B virus (HBV) [151]. Studies have shown that CD4^+^ and CD8^+^ T cells of patients infected with *P. falciparum* express PD-1 to a large extent [152]. This action can contribute to the immune evasion mechanism induced by *P. falciparum* [153]. Also, the infection of immune cells with HIV leads to an increase in the expression of ICPs such as CTLA-4, PD-1, LAG-3, and TIM-3, which disrupts the functions of NK cells and CTLs leads to preventing the removal of virus-infected cells [153, 154]. Therefore, ICPs can act as a double-edged sword based on which cells are expressed and at which stage of immune cells development ICPs interact with them [155].

During the past decades, due to the role defined for ICPs in the pathogenesis of infectious diseases and cancer, blocking the function of these molecules has been proposed to treat these diseases. In many studies, antibody-based immune checkpoint blockades (ICBs) have been used to inhibit the function of ICPs and have had promising results [156, 157]. However, it seems that blocking the function of one of the ICPs leads to a compensatory increase in the expression of other ICPs on the surface of tumor and infected cells [158, 159]. Table 3 summarizes 10 FDA-approved ICBs that are used in different types of cancers. It has also been shown that drugs such as metformin [160], curcumin [161], etoposide [162], etc., can affect the expression of ICPs and their ligands. In addition, nanobodies [163, 164] and small molecules [165] have also been used to block the functions of ICPs.

**Table 3:** FDA-approved immune checkpoint inhibitors with approved indications

No	Drug Name	Generic Name	Target	Date of Approval	Applications
1	Ipilimumab	Yervoy®	CTLA-4	2011	1. Melanoma 2. Renal cell carcinoma 3. CRC 4. HCC 5. NSCC 6. Malignant pleural mesothelioma 7. Esophageal cancer
2	Tremelimumab	Imjudo®	CTLA-4	2022	1. HCC 2. Mesothelioma
3	Pembrolizumab	Keytruda®	PD-1	2014	1. NSCC 2. HCC 3. CSCC 4. RCC 5. HNSCC 6. Urothelial carcinoma 7. NMIBC 8. Colorectal cancer 9. gastric cancer 10. Esophageal cancer 11. Cervical cancer 12. Merkel cell carcinoma 13. Endometrial carcinoma 14. Classical Hodgkin lymphoma 15. Primary mediastinal large B-cell lymphoma 16. Triple-negative breast cancer
4	Nivolumab	Opdivo®	PD-1	2014	1. NSCC 2. Melanoma 3. RCC 4. malignant pleural mesothelioma 5. Classical Hodgkin lymphoma 6. SCC 7. Urothelial carcinoma 8. Colorectal cancer 9. HCC 10 Esophageal cancer 11. Gastric cancer 12. Gastroesophageal junction cancer 13. Esophageal adenocarcinoma
5	Cemiplimab	Libtayo®	PD-1	2019	1. SCC 2. BCC 3. NSCC
6	Dostarlimab	Jemperli®	PD-1	2021	Endometrial cancer
7	Durvalumab	Imfinzi®	PD-L1	2017	1. NSCC 2. SCLC 3. HCC 4. Biliary tract cancer
8	Atezolizumab	Tecentriq®	PD-L1	2016	1. HCC 2. NSCC 3. SCLC 4. ASPS 5. Melanoma
9	Avelumab	Bavencio®	PD-L1	2017	1. Urothelial carcinoma 2. MCC 3. RCC
10	Relatlimab	Opdualag®	LAG-3	2022	Melanoma

*NSCC* Non-small cell lung cancer, *SCC* Cutaneous squamous cell carcinoma, *BCS* Basal cell carcinoma, *MCC* Merkel cell carcinoma, *RCC* Renal cell carcinoma, *SCLC* Small cell lung cancer, *CRC* Colorectal cancer, *HCC* Hepatocellular carcinoma, *ASPS* Alveolar soft part sarcoma, *CSCC* Cutaneous squamous cell carcinoma, *HNSCC* Head and neck squamous cell carcinoma, *NMIBC* Non-muscle invasive bladder cancer

However, as we said, the function of ICPs in physiological conditions is essential for health, and defects in the expression of these molecules can lead to various autoimmunities [166]. Based on this and considering the immunomodulatory role of MSCs, it is suggested that ICPs have a vital role in the therapeutic potential and immunosuppression induced by these cells. Various studies have shown that MSCs express different types of ICPs and their ligands and thus can influence the responses of T cells, macrophages, NK cells, and other innate and adaptive immune system cells. Also, the expression of ICPs affects the MSCs' regenerative potential and migration ability. In addition, knowing the ICPs of MSCs can help to decide on their selection as an appropriate treatment option for various diseases.

## Cytotoxic T-lymphocyte associated protein 4 (CTLA-4) expression by MSCs

CTLA-4, one of the most important immune checkpoints expressed on immune system regulatory cells, including Treg cells, plays an important role in immunomodulation [167]. A defect or mutation in the expression of this molecule can lead to inflammatory responses and various autoimmune diseases [168–170]. New studies have shown that MSCs can also express CTLA-4 and thus play a role in immune regulation [171]. Studies show that cells express different isoforms of CTLA-4 in different conditions. Also, CTLA-4 expressed by MSCs through alternating splicing can be in the form of 4 isoforms [171], which include 1) the full-length version (flCTLA-4) that has all the regions related to binding to the ligand, transmembrane, transduction intracytoplasmic domain [172], 2) the type that lacks the ligand binding domain (liCTLA-4), 3) lacks the transmembrane domain (sCTLA-4) and is secreted into the extracellular environment, and the fourth type that lacks both ligand binding and transmembrane domain (1/4 of CTLA-4) [172].

Further investigations of MSCs through qPCR analysis show that the expression level of sCTLA-4 is higher than that of flCTLA-4 [171]. The critical point is that hypoxia can increase the expression of sCTLA-4 in MSCs [171]. sCTLA-4 produced by MSCs can be detected in the supernatant, and the therapeutic uses of the MSCs-derived supernatant [173] play an essential role in induced anti-inflammatory responses [85]. In a study, it has been shown that the addition of anti-CTLA4 antibody to the coculture of MSCs and PBMCs stimulated with phytohemagglutinin (PHA), both in hypoxic and normoxic conditions, leads to the suppression of anti-inflammatory responses induced by MSCs [171]. These results show the importance of sCTLA-4 and flCTLA-4 associated with MSCs in their anti-inflammatory responses.

Another study showed that anti-CTLA-4 antibodies could not reverse the MSCs-induced anergy in T cells [174]. Therefore, it seems this type of anergy, which results from the co-culture of T cells with MSCs, is through a pathway independent of CTLA-4 [175]. Also, in another study, this issue has been confirmed and shown that MSCs independent of CTLA-4 increase the frequency and differentiation of Treg and lead to the reduction of Th17 cells [176].

## Programmed cell death ligand (PD-L) expression by MSCs

Programmed cell death-1 (PD-1) and its ligands PD-L1 and PD-L2 are crucial in controlling immune responses in the hemostasis phase. PD-L1 and PD-L2 are expressed on the surface of tumor cells and lead to immune deviation [177]. MSCs express PD-1, PD-L1, and PD-L2 and bind to their ligands on the surface of B cells, T-cells, and other cells [178]. PD-1/PD-L1 interaction can suppress T cell functions through different mechanisms. One of these mechanisms is the suppression of proliferation in T cells [179]. As mentioned in various studies, the presence of IFN-γ can lead to an increase in the immunosuppressive ability of MSCs, which does this by increasing the expression of PD-L1. According to new studies, it has been shown that binding of IFN-γ to the IFN-γR in MSCs leads to the activation of the JAK/STAT1/IRF1 pathway, and by binding the IRF1 to the PD-L1 promoter increases its expression. On the other hand, TNF-α, as another pro-inflammatory cytokine, does this by activating the NF-κB transcription factor. The important point is that TNF-α alone is not able to increase the expression of PD-L1, and it does this effect synergistically in combination with IFN-γ. In this way, the NF-κB transcription factor helps in this process by increasing the expression of IFN-γR by MSCs [180].

In such a way that in the case of MSCs culture in the presence of anti-PD-L1 siRNA or MSCs co-culture with active lymphocytes derived from IFNγ^−/−^, it cannot stimulate the suppressive function of MSCs [181]. Polyinosinic-polycytidylic acid (polyI:C), as a synthetic ligand of Toll-like receptor 3 (TLR3), increases PD-L1 expression in tonsil-derived MSCs [182]. The results of a study that used Poly I:C pretreated MSCs show that their co-culture with T cells isolated from the spleen leads to the suppression of the differentiation of naive T cells into Th1, Th2, and Th17 [182]. However, this inhibition seems to be stronger for Th17 than others. Based on the results of this study, when MSCs are used for their immunomodulatory properties, manipulating them, such as adding Poly I:C, can increase these properties and achieve better treatment results. As we know, MSCs reduce the differentiation of inflammatory cells and improve the differentiation and function of Treg cells [183]. The results of Fei Gao et al.'s study showed that MSCs perform this action at least partially by expressing PD-L1 and by inhibiting the Akt/mTOR signaling pathway [184]. Because the use of siRNA against PD-L1 can suppress the function of MSCs in stimulating Treg differentiation to some extent. The use of MSCs expressing PD-L1 in TNBS-induced colitis rats leads to the improvement of disease symptoms [184]. Also, PD-1/PD-L1 interaction between adipose tissue-derived MSCs and PBMCs can suppress TCD8^+^ and TCD4^+^ cell responses through the negative regulation of NF-κB function [185]. The results of the study conducted by Kaijian Zhou et al. show that the co-culture of MSCs and PBMCs significantly reduces the phosphorylation of NF-κB, which is a critical step in the migration of this factor to the nucleus for transcription, and this action is in the presence of anti-PD- L1 antibodies is inhibited [185].

New studies also confirm the results of previous studies. In a study conducted by Rosanna Di Tincoet et al. in 2021, it has been shown that the co-culture of MSCs derived from dental pulp with PBMCs can lead to a significant decrease in mRNA levels of IL-10, IFNγ, CXCL10, TNF-α, IL-2, and CCL5 [186]. This co-culture also led to a significant increase in PD-L1 expression in MSCs. However, the study's results show that using PD-L1 antibodies and their blockade does not lead to losing the immunosuppressive effect of MSCs [187]. Therefore, it is suggested that MSCs use alternative methods to suppress the immune response. In this study, it has been shown that the PD1/PD-L1 pathway coordinates with the Fas/FasL pathway by increasing the expression of FasL (in the presence of PD-L1 blockade) by MSCs to modulate immune system responses in PBMCs [188]. PD-L1 expressed by MSCs modulates the responses of PBMCs but also helps MSCs maintain immunomodulatory properties [186]. In addition to the expression of PD-L1 on the surface of MSCs, these cells can secrete PD-L1 into the extracellular environment. Inflammatory licensing for MSCs through an N-glycosylation-dependent post-translational regulatory mechanism leads to increased expression and secretion of PD-L1 by MSCs [189]. Also, the level of PD-L1 expression has a strong relationship with their therapeutic and immunomodulatory potentials in the mouse model of autoimmune hepatitis [190].

**Fig. 1:**
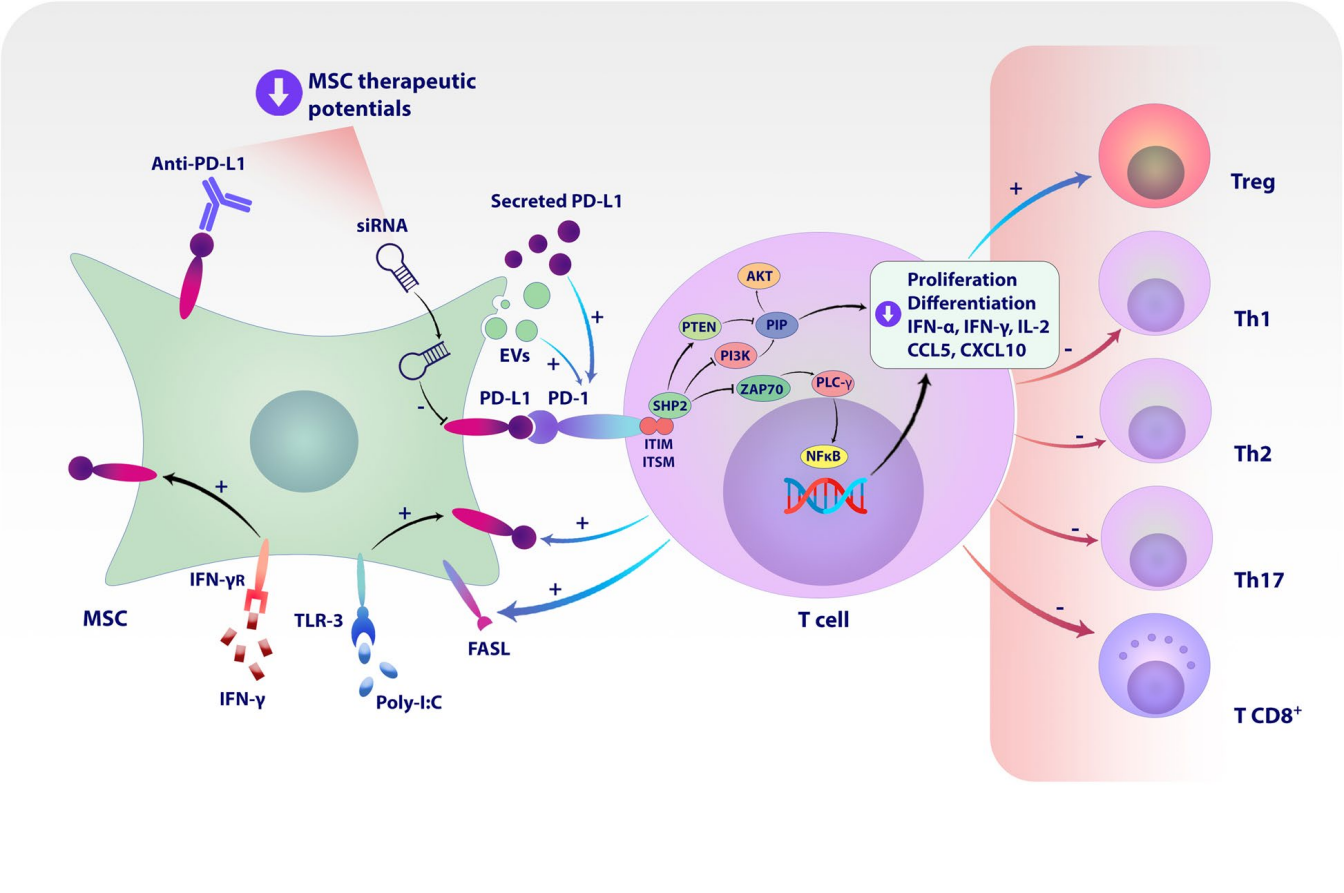
PD-L1 mediated immunomodulatory and therapeutic potential of mesenchymal stromal/stem cells (MSCs). PD-L1 expressed by MSCs can interact with PD1 on the surface of T cells in three mechanisms. In the first option, PD-L1 is transferred to the cell plasma membrane and, in this way, inhibits the functions of T cells through direct cell-to-cell interaction. In the second mechanism, PD-L1 is secreted into the extracellular environment. For this reason, MSCs-derived supernatant can induce PD-L1-mediated immunomodulatory functions in co-culture with T cells or PBMCs. In the third method, MSCs affect the response of T cells by transferring PD-L1 to extracellular vesicles (EVs) such as exosomes. The interaction between PD-1 and PD-L1 leads to the creation of signaling cascades in T cells, which inhibits their proliferation and differentiation into inflammatory populations, including Th1, Th2, and Th17, as well as the production of inflammatory cytokines. It has also been found that this interaction also inhibits the function of TCD8 + cells. However, this interaction increases the differentiation of T cells towards regulatory T cells. In addition, it has been shown that signal transmission from PD-1 leads to positive feedback to PD-L1^+^ MSCs and enhances their immunomodulatory functions. It has also been shown that the use of inflammatory environments and pretreatment of MSCs with TLR ligands, including poly I:C, can lead to an increase in the immunomodulatory potential of MSCs by increasing the expression and production of PD-L1. This is while the use of anti-PD-L1 antibodies and siRNA inhibiting its expression and translation can reduce the immunomodulatory potential of MSCs. Therefore, the expression of PD-L1 plays an essential role in the immunomodulatory potential of MSCs, which leads to an increase in its therapeutic potential in autoimmune colitis and psoriasis animal models

It is also possible that in addition to PD-L1 expressed on the surface and secreted into the extracellular environment by MSCs, this molecule is transferred to the target cell by small extracellular vesicles (sEVs) and exerts its immunoinhibitory function. Studies show that in patients with aGvHD, the amount of PD-L1 containing sEV in blood plasma increases after infusion of Wharton jelly-derived MSCs. It has also been shown that the amount of this sEV is entirely related to the time of injection, and 30 min after the injection, the increase in the amount of PD-L1 containing sEV can be evaluated (Fig. 1). These extracellular vesicles can suppress T-cell responses in a TCR-dependent manner. Meanwhile, using Wharton's jelly MSCs genetically modified by the CRISPR/CAS9 system to not express PD-L1 produces sEVs that cannot suppress T-cell responses [191]. Finally, it has been shown that the use of Wharton jelly derived-MSCs can lead to the improvement of patients with aGvHD symptoms and conditions. This study also reports that, in addition to the fact that, like previous studies [192], IFNγ increases the expression of PD-L1 by MSCs but also leads to an increase in the secretion of sEV-PD-L1 from these cells [193].

## Inducible costimulator ligand (ICOSL) expression by MSCs

ICOSL is a member of the B7 family and plays a vital role in follicular helper T cell interaction and high-affinity antibody production [194]. According to the results of the studies, it has been shown that blocking the ICOS-ICOSL interaction aggravates the experimentally induced allergic encephalomyelitis in the model [195]. Therefore, the inhibitory signaling resulting from this interaction seems to negatively affect the immune responses. ICOS is expressed by various types of cells, including tumor cells, antigen-presenting cells, and epithelial cells, and is very important for the function of Treg cells [196, 197]. Studies have shown that placing MSCs in inflammatory conditions leads to increased expression of ICOSL by these cells [198].

The results of the study conducted by Lee et al. show that, in addition to other mechanisms, the co-culture of MSCs with T cells increases the differentiation of Treg cells through the ICOS-ICOSL interaction-dependent pathway. It has also been shown that blocking ICOSL expressed on the surface of MSCs reduces their ability to induce Treg cell responses [199].

**Fig. 2:**
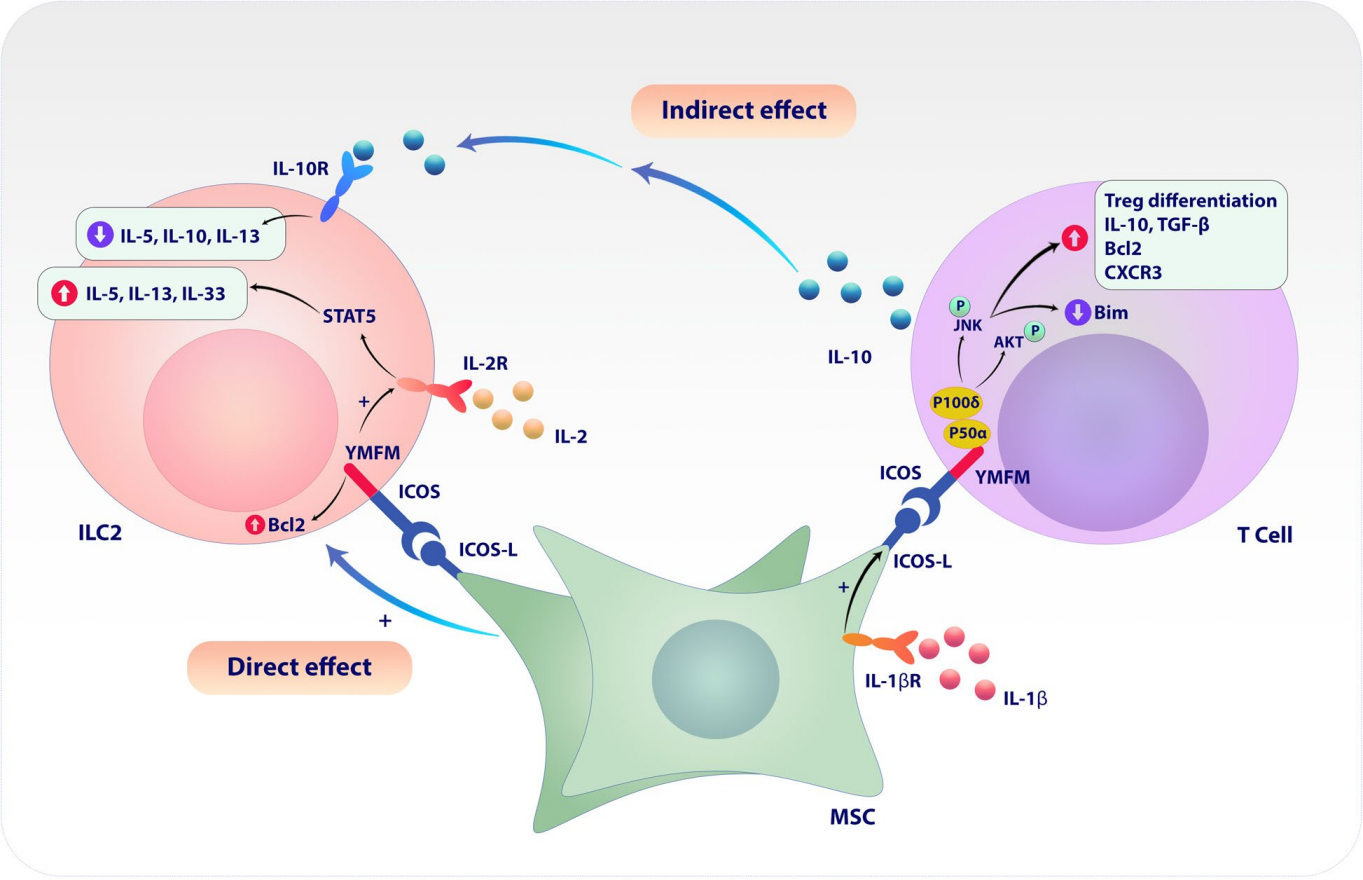
ICOSL mediated immunomodulatory of mesenchymal stromal/stem cells (MSCs) on T cells and ILC2. MSCs express a low amount of ICOSL in the naive state. However, the co-culture of MSCs by TCD4^+^ cells in the regulatory T cell-inducing conditions increases ICOSL expression up to tenfold according to qPCR results. Also, this co-culture leads to an increase in the number and percentage of regulatory T cells compared to the culture of TCD4.^+^ cells only under regulatory induction conditions (as the control group). It has been shown that the co-culture of MSCs with PBMCs also leads to the expansion and function of the regulatory T-cell population. The effect of MSCs on ILC2 functions is direct and indirect. In indirect conditions, the co-culture of MSCs with PBMCs suppresses the functions of ILC2 by expanding the population of regulatory T cells and their IL-10 production. Meanwhile, the direct co-cultivation of ILC2 with MSCs increases the functions of ILC2 through ICOS-ICOSL interaction. ILC-2 that have been directly co-cultured with MSCs have a high survival rate and highly produce cytokines such as IL-5, IL-10, and IL-33

Another study conducted in 2021 showed that the use and coculture of MSCs with PBMCs leads to the inhibition of type 2 responses by inhibiting the differentiation of Th2 cells and type 2 innate lymphoid cells (ILC2) [200]. A significant point about these cells is that the direct cell-to-cell contact of MSCs with ILC2 mediated by ICOS-ICOSL interaction leads to increased ILC2 activity [201]. The results show that MSCs exert their inhibitory effects on ILC2 functions through the induction of Treg cells [201]. Tregs alone cannot inhibit the responses of ILC2s, but after co-culture with MSCs, they acquire this ability [201]. Further investigations to find the mechanism of MSCs' influence on the suppressive responses of Tregs show that ICOS-ICOSL interaction is one of the main factors [201]. Therefore, it was demonstrated that the co-culture of Tregs with MSCs through ICOS-ICOSL interaction increases the ability of Tregs to suppress the functions of ILC2 and leads to a significant decrease in the production of IL-13, IL-9, and IL-5 cytokines by ILC2 [201]. Studies show that Tregs do this by producing IL-10 induced by ICOS-ICOSL interaction [201] (Fig. 2).

## CD39 and CD73 expression by MSCs

As we know, adenosine is one of the suppressors of the immune system, which performs its function by binding to the A2A receptor (ADORA2A) [202]. Extracellular adenosine is usually produced from ATP by two molecules, CD39 and CD73 [203]. During this process, the adenosine deaminase converts adenosine into inosine [204]. Extracellular adenosine can suppress the proliferation and responses of T cells [205]. The results of various studies have shown that the co-culture of T cells with MSCs can suppress T cell responses [9, 60]. Therefore, considering the importance of this molecule (CD39) in T-cell responses, the researchers investigated the expression of CD39 on the surface of MSCs and its effect on modulating immunity induced by MSCs. Various methods, including flow cytometry, have shown that CD39 has a permanent expression on the surface of MSCs [206]. However, when MSCs are exposed to inflammatory conditions, the level of CD39 expression increases from 15 to 35%. Also, the amount of adenosine production, evaluated by high-pressure liquid chromatography (HPLC), was associated with an increase of 2 times in co-culture with activated T cells [206]. Therefore, in inflammatory conditions, these cells can produce more adenosine and suppress the immune system's responses more strongly. It has been shown that the co-culture of human MSCs with activated T cells leads to a threefold increase in MSCs double positive for CD39 and CD73 [206]. On the other hand, this co-culture affects the responses of T cells and is associated with a significant increase in ADORA2A in these cells, which is associated with a decrease in the proliferative activity of these cells [206]. Also, the use of ADORA2A antagonist (ZM 241385) [207] in this co-culture system led to a significant increase in proliferation in T cells, which shows the importance of adenosine in suppressing the proliferative responses of T cells [206].

Mouse studies have also shown that CD39 and CD73 are simultaneously expressed on the surface of MSCs and perform essential inhibitory functions on the immune system [208]. Many studies have suggested that soluble factors produced and secreted by MSCs, including TGF-β and HGF, lead to decreased proliferation in activated T cells [209, 210]. However, the study conducted by Sattler et al. showed that using monoclonal antibodies against HGF and TGF-β receptors does not significantly increase T cell proliferation [208]. Also, kynurenine, a tryptophan metabolite through the action of IDO enzyme [211], was not observed in the MSC supernatant. Therefore, they proposed another factor responsible for this proliferation suppression [208]. CD39 expressed on the surface of MSCs leads to the production of adenosine, suppressing the proliferation of T cells [208]. It has been shown that the use of SCH58261, an antagonist of ADORA2A [212], and the use of polyoxotungstate 1 (POM-1) as a CD39 inhibitor [213] separately lead to the reversal of the suppression induced by MSCs in coculture conditions [208].

In addition, MSCs can suppress the function and differentiation of Th17 cells through CD39 [214]. Co-cultivation of T cells with MSCs leads to a decrease in the production of IL-17A/IFN-γ and an increase in the expression of CD39 and CD73 on the surface of T cells [214]. Using monoclonal antibodies against CD39 can significantly reduce the inhibition applied to Th17 differentiation. So, it can be concluded that MSCs, through a CD39-dependent pathway, inhibit Th17 function and proliferation [214]. Also, mass spectrometry analysis showed that the amount of adenosine in the supernatant increases during the co-culture of Th17 cells and bone marrow-derived MSCs [214]. This is while the use of monoclonal antibodies against CD39 leads to a significant decrease in the production of adenosine and its amount in the supernatant of the coculture system.

Considering the importance of the expression of these molecules, in the study conducted by Tan et al., it was shown that MSCs isolated from C57BL/6 mice adipose tissue include two populations in terms of CD73 expression. One of these populations expresses CD73 at a low level (CD73^low^), and the other expresses a high level of CD73 (CD73^high^) [215]. These cells differ from each other in terms of function and therapeutic potential. Examining the ability of these cells to repair myocardial infarction (MI) damages in a model murine induced by 2OA-BSA [216] and transplanting both MSCs subpopulations show that the CD73^high^ subpopulation has a higher ability to repair and can lead to improving the structure and function of the heart [215]. The transplantation of these two types of cells seems to have no difference in the amount and the type of recruited immune cell population to the MI heart tissue. However, the results of the study show that the CD73^high^ subpopulation leads to the reduction of inflammation and the modulation of immune system responses through the positive regulation of the expression and production of several anti-inflammatory cytokines, including IL-4 and IL-10, and the reduction of the expression and production of inflammatory cytokines such as TNF-α. In addition to the effect on cytokines, these cells affect the expression of other anti-inflammatory molecules, including TGM-2 and arginase-1 (Arg-1), and reduce the expression of NOS2 [215]. Therefore, it seems that the immunomodulatory efficiency of CD73^high^ MSCs is higher than the CD73^low^ subpopulation. In this way, it helps to improve the functions and prevent damaging inflammations to the heart tissue after MI. The results of the in vitro studies also show that compared to the CD73^low^ subpopulation, CD73^high^ MSCs significantly lead to the differentiation of macrophages to the M2 phenotype and functions related to the regeneration of damaged tissues [215]. The importance of the effect of the expression of these molecules and the axis induced by them, that is, CD39/CD73/adenosine, in the therapeutic potential of MSCs in other diseases has also been investigated. For example, in a study where MSCs were used to treat autoimmune arthritis, the role of CD39/CD73/adenosine was evaluated [217]. Molecular studies show that MSCs reduce the expression of NF-kB and p65/p50 in vitro conditions and lead to decreased osteoclastogenesis [218]. Also, studies showed that transplantation of MSCs into autoimmune arthritis DBA/1J mice model led to decreased RANKL expression in synovial tissue and osteoclast formation [217]. Using inhibitors and blocking the function of CD39 through POM1, blocking CD73 by APCP, or inhibiting the functions of adenosine by inhibiting its receptor, i.e., adenosine A2A receptor inhibitor (SCH58261) or adenosine A2B receptor inhibitor (Alloxazine), lead to reverted treatment outcome induced by MSCs [217].

**Fig. 3:**
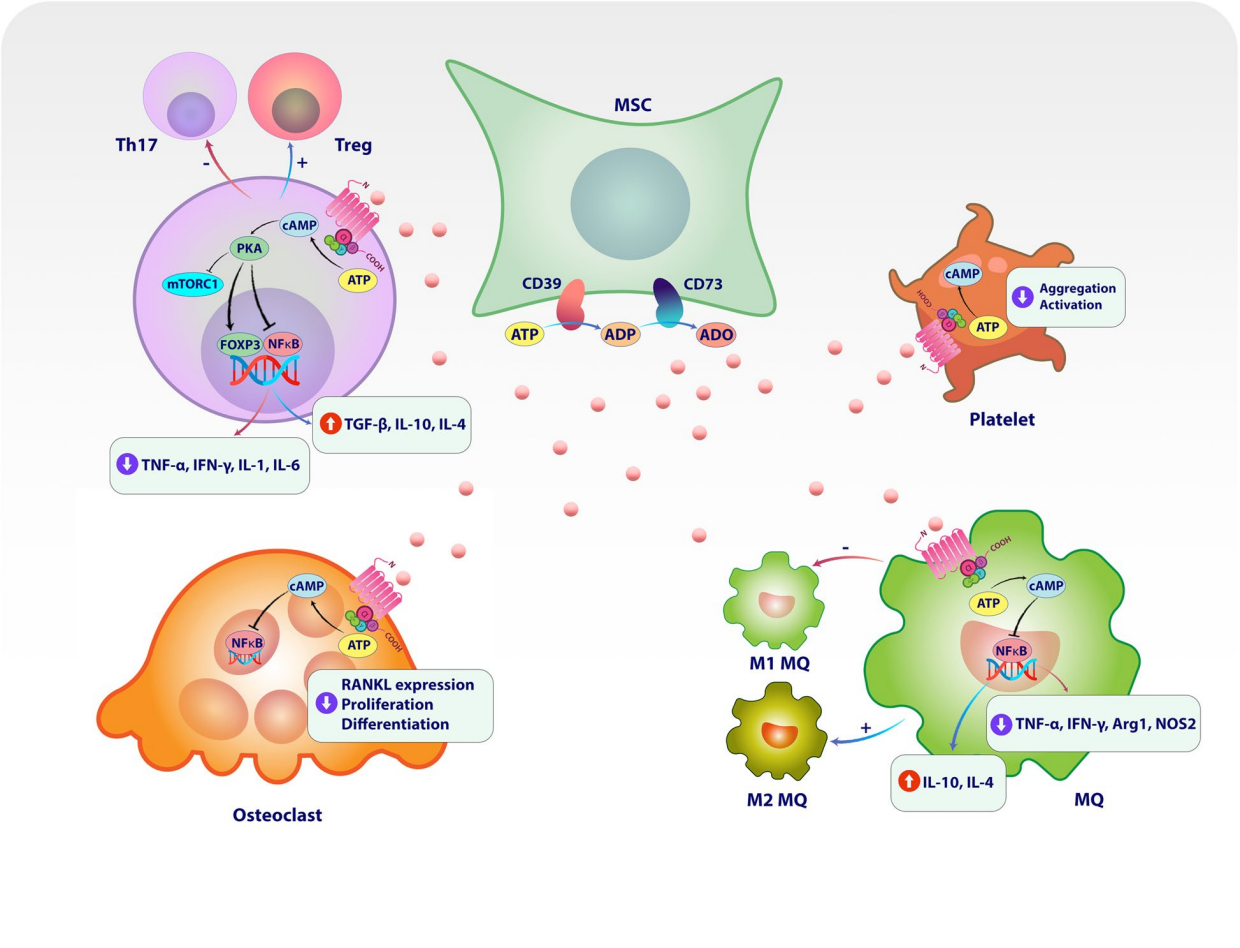
CD39, CD73, and adenosine mediated immunomodulatory of mesenchymal stromal/stem cells (MSCs) on T cells, macrophages, and osteoclasts. Extracellular ATP is first converted to ADP and then to AMP through CD39. The produced AMP is used as a substrate for the function of CD73 and leads to the production of adenosine. Adenosine receptor (A2AR) can be expressed on the surface of various cells, including immune cells and tumor cells. The binding of extracellular-produced adenosine to A2AR on the surface of tumor cells can increase their survival and proliferation of tumor cells and facilitate epithelial-mesenchymal transition (EMT). Also, its binding to immune cells expressed A2AR can lead to reduced survival and production of inflammatory cytokines by inhibiting pathways related to mTORC and NF-κB. It seems that tumor cells, by expressing CD39 and CD73, help their immune escape. MSCs also express CD39 and CD73, and by producing adenosine, they affect the functions of immune cells, including T cells, macrophages, and osteoclasts. Adenosine produced by MSCs inhibits the functions of Th17 and M1 macrophages while they increase the functions of regulatory T cells and M2 macrophages. Adenosine can also reduce the function and proliferation of osteoclasts. It has also been shown that MSCs that produce adenosine can reduce the aggregation and activation of platelets

In addition to the role of markers such as CD73 and CD39 in modulating immune responses induced by MSCs, these molecules also perform other functions. Studies show that MSCs have procoagulant and anticoagulant activity [219], but their supernatant showed to cannot do phenomenon [220]. Therefore, MSCs perform this action through cell–cell interaction. It has also been shown that this inhibition of activation is independent of the molecules related to the activation of platelets, i.e., P-selectin and cyclooxygenase [220]. The study conducted by P. Netsch and his colleagues shows that MSCs isolated from different sources do this through the CD73-produced adenosine-dependent mechanism and lead to preventing the platelets activation and their aggregation. In this study, the use of adenosine deaminase (ADA), which converts adenosine to inosine [221], led to the removal of the inhibition induced by MSCs, which confirms the role of CD73 in inhibiting platelet activation [220] (Fig. 3).

## Galectins expression by MSCs

Galectins are a group of molecules related to lectins that exert their biological effects by binding to galactoside (in the receptor's structure) [222]. The results of the studies have shown that this ligand, by binding to its receptor, T cell immunoglobulin and mucin domain-containing protein 3 (TIM3), performs actions such as homeostasis of immune system responses [223]. Studies have shown that treating diseases such as autoimmune disorders and patients who have received allograft transplants leads to the improvement of the patients and increases the survival of the transplanted tissue [224]. TIM3 plays an important role in suppressing the responses of Th1 cells and prevents the production of inflammatory cytokines such as IFN-γ and TNF-α [225]. Also, the interaction of galectin-9 (Gal-9) with TIM3 leads to suppressing the functions of Th17 and cytotoxic T cells [226].

Various studies show that MSCs suppress immune system responses through Gal-9 production and have a role in their therapeutic potential. MSCs culture in IFN-γ containing medium leads to increased production of soluble Gal-9 by MSCs [227]. Therefore, it can be concluded that the production of Gal-9 from MSCs depends on the STAT and JNK signaling pathway. A study published in 2018 showed that the transplanted MSCs suppress the proliferation and differentiation of Th1 and Th17, leading to the reduction of liver inflammation in the autoimmune cholangitis mice model [228]. After transplanting MSCs to autoimmune cholangitis model mice, the level of Gal-9 in the liver and serum increases significantly. Since Gal-9 is secreted by MSCs, the supernatant of these cells can perform Gal-9-mediated therapeutic and immune suppression functions [228]. The use of α-lactose to inhibit the function of Gal-9 in MSCs-CM leads to the reduction of the therapeutic potential and reverses the suppression of the proliferation of CD4^+^ T cells. Therefore, it is suggested that the soluble Gal-9 produced by MSCs is one of the immunosuppressive mechanisms mediated by these cells. It has also been shown that the therapeutic potential of MSCs that express high levels of Gal-9 is significantly higher than Gal-9 blocking MSCs in endotoxemia induced by LPS [229]. Examining the spleen cells of Gal-9 highly expressed MSCs recipients shows that the amount of M2 macrophages and Treg in them is associated with a significant increase compared to other groups [229]. The therapeutic use of MSCs in septic mice has also shown that these cells can improve kidney functions in septic mice through Gal-9 production and Th1/Th2 balance adjustment. Also, the results of this study showed that the use of MSCs can affect the Th17/Treg axis by TIM3/Gal-9 interaction. Considering that the use of whole-soluble TIM3 can reverse the therapeutic effects of MSCs, it seems that Gal-9 is one of the effective immunomodulatory factors produced by these cells [230].

Another study showed that MSCs lead to the differentiation of tolerogenic DCs through the production of Gal-1 [231]. Co-cultivation of MSCs with DCs leads to a decrease in the expression of MHC-II and co-stimulatory molecules such as CD80, CD83, and CD86 on the surface of DCs. Also, this co-culture leads to an increase in the production of IL-10, IL-12, and Gal-1 in the supernatant. Further investigations show that Gal-1 produced from MSCs through inhibiting the p38 MAPK signaling pathway leads to suppression of proliferation and increased anti-inflammatory activity in DCs [231]. In addition, the study conducted by Yoojin Seo et al. showed that in the co-culture system, MSCs that produced Gal-1 to the supernatant can prevent the differentiation of microglia to M1 pro-inflammatory phenotype. This function of MSCs is suppressed through a selective Gal-1 inhibitor (OTX008) [232]. Also, MSCs can affect the alloreactive CD4^+^ and CD8^+^ T cell responses through the production of Gal-1 and inhibit their function. The result of the study conducted by Gieseke et al. shows that MSCs can reduce the proliferation of CD4^+^ T cells in a Gal-1-dependent manner. However, MSCs produced Gal-1 do not seem to play a role in modulating NK cell responses [233].

Therefore, it seems that MSCs produced Gal-1 play an important role in their immunomodulatory potential by affecting DCs, MQ, and T cell functions.

## CD155 (Poliovirus receptor) expression by MSCs

CD155, also known as poliovirus receptor (PVR), is a ligand expressed on the surface of different cells, and its receptor expressed on the surface of T and NK cells called T cell immunoreceptor with immunoglobulin and ITIM domain (TIGIT) [234]. The interaction of CD155 with TIGIT leads to the initiating of inhibitory responses in TIGIT-expressing cells. For example, this interaction leads to a decrease in cytotoxic activity, the production of cytokines, and a reduction in the degranulation of NK cells [235]. In addition, CD226 (DNAM-1) can also bind to CD155, but the affinity of TIGIT for CD155 is higher than for CD226 [236, 237]. Also, new studies have shown that CD155 is highly expressed on the surface of MSCs and can be responsible for a part of the immune inhibition induced by these cells. NK cells express both TIGIT and CD226, which are CD155 receptors [238]. The binding of CD155 to CD226 leads to increased activity of NK cells, and its binding to TIGIT leads to functional inhibition of T cells [239]. As mentioned, the affinity of TIGIT to CD155 is higher than the binding affinity of CD226 to CD155, and therefore, due to the possible simultaneous involvement of both inhibitory and stimulatory receptors in the presence of CD155, the function of the inhibitory receptor is overcome and leads to the suppression of NK cell responses [237, 240].

The results of the studies show that in myelodysplastic syndrome (MDS), the activity of NK cells becomes abnormal, and a decrease accompanies the antibody-dependent cytotoxic activity and cytolytic activity [241, 242]. Considering the presence of MSCs in the bone marrow, it seems that these cells play a role in the progression of the disease. MSCs affect the expression and secretion of factors related to hematopoiesis and play a role in immune regulation by producing cytokines, growth hormones, intercellular communication, and exosomes [9]. The results show that the culture of MSCs in inflammatory conditions increases the amount of CD155 on the surface of these cells [243]. However, the exact mechanism of how MSCs work in different diseases is different, and depending on the type of tumor and disease, they may use different main mechanisms. Investigations to search for ligands related to NK inhibitory receptors, including CD155, CD112, and CD113, show that the expression of CD155 on the surface of MDS patients isolated MSCs increased significantly compared to MSCs isolated from healthy controls [244]. It has also been shown that blocking TIGIT and activating CD226 can reverse the inhibitory effect induced by MSCs on NK cells. Therefore, the in vitro results indicate that MSCs can suppress the inflammatory functions of NK cells through the CD155/TIGIT pathway [244, 245]. It is also known that the CD155/TIGIT pathway can lead to the exhaustion of NK cells [246]. The results of examining NK cells isolated from multiple myeloma (MM) patients also show that these cells have exhausting markers, and the level of TIGIT in them is associated with an increase compared to healthy control [234, 247]. In addition, MSCs isolated from multiple myeloma patients also have a significant increase in CD155 expression. The in vitro results conducted by Yun Liu et al. show that the co-culture of NK cells with MSCs leads to CD155/TIGIT interaction and induction of exhaustion in NK cells [234]. So, it proposed that blocking TIGIT can restore NK cell exhaustion and provide a potential avenue for antitumor immunotherapy for multiple myeloma patients.

## Herpes virus entry mediator (HVEM) expression by MSCs

HVEM is another ligand that binds to immune checkpoints family family-related receptors [248]. The receptor of this molecule (HVEM) is B and T Lymphocyte attenuator (BTLA), which binds to it and inhibits the functions of lymphocytes [249]. BTLA has been identified as the third immune checkpoint after PD-1 and CTLA-4, and having 2 ITIM motifs leads to the calling of SHP2, which inhibits signaling events related to inflammatory responses [250, 251]. HVEM is expressed by various types of tumor cells, including melanoma cells, and in this way, they suppress the antitumor responses of CD8^+^ T cells [252]. It has also been shown that HVEM has a broader expression than PD-L1 in melanoma cells, and its expression level is associated with a poor prognosis [252, 253]. Therefore, it is imperative to investigate the cells that express this molecule and its functions.

The results of published studies have shown that HVEM is highly expressed on the surface of MSCs and is responsible for part of the anti-inflammatory functions of MSCs in treating inflammatory diseases [254]. In LPS-stimulated mice, alveolar macrophages have been shown to accumulate near lung tissue-resident MSCs (LRMSCs) [255]. Therefore, LRMSCs may be responsible for inducing anti-inflammatory responses in these macrophages. The results of RNA sequencing obtained from co-culture of LRMSCs with alveolar macrophages incubated with LPS show the downregulation of the expression of molecules involved in various inflammatory signaling pathways related to TLRs, TNF, JAK-STAT and PI3K-Akt in them [255]. Also, as a result, the level of expression of inflammatory cytokines in alveolar macrophages decreases. Also, the co-culture of LRMSCs with splenocytes in LPS-stimulated conditions reduces inflammatory responses [255]. Therefore, LRMSCs can suppress the production of inflammatory cytokines from innate and adaptive immune cells. Injection of LRMSCs into LPS-induced ARDS model rats also had similar results to in vitro. Considering the importance of HVEM, its expression level in LRMSCs isolated from LPS-induced ARDS model rats was five times higher than in LRMSCs isolated from the lungs of the sham group [255]. Further studies showed that MSCs overexpressing HVEM have a greater ability to suppress immune cell responses than MSCs with low HVEM expression. It has also been shown that the expression of BTLA on the surface of immune cells is required to induce the increased anti-inflammatory response of MSCs by HVEM.

HVEM expression has also been observed in MSCs isolated from other tumor tissues. Considering that MSCs are present in the tumor microenvironment as one of the cell populations [256], it has been shown that these cells play an important role in the chemoresistance of tumor cells in intrahepatic cholangiocarcinoma (ICC) [257]. Compared to other tissues (for example, umbilical cord-derived MSCs), MSCs isolated from ICC tissue have a high level of HVEM expression. In this way, they help the survival of tumor cells and prevent their apoptosis [257]. MSCs activate AMPK/mTOR-mediated autophagy in cholangiocarcinoma cells by overexpressing HVEM and producing IL-6 [257, 258].

## Tumor necrosis factor receptor 2 (TNFR2)

As a pro-inflammatory cytokine in soluble and membrane-bound forms [259], TNF-α plays an important role in forming immune system responses [260]. This cytokine has two separate receptors, including TNFR1 and TNFR2 [261]. However, it seems that the affinity of TNFR1 for both soluble and membrane-bound forms of TNF-α is higher than TNFR2 [262, 263]. TNFR1 has a wide expression on different cells [264]. After binding to TNF-α, it can increase proliferation, differentiation, and survival by activating signaling pathways related to NF-kB and MAPK or by using the death domain (DD) and activating related RIP1-dependent and RIP1-independent leads to the activation of caspase 8 and the initiation of apoptosis in cells [263, 265]. It seems that the choice between whether the cell with TNFR1 receptor after binding to TNF-α is selected for survival or apoptosis depends on the responding cell cellular stress and metabolic state [266]. Although TNFR1, TNFR2 is expressed in some cells, including immune cells, endothelial cells, neurons, and MSCs [265]. TNFR2 signaling leads to the proliferation and differentiation of Treg cells as well as the proliferation and survival of tumor cells [267, 268]. The results of the studies have shown that antibodies against TNFR2 can be a potential treatment for patients with ovarian cancer by inducing Treg cells and apoptosis in tumor cells [269]. Therefore, today, TNFR2 is called as one of the emerging immune checkpoints [270]. Considering the therapeutic role of immunomodulatory functions in MSCs-mediated treatments, it has been shown that TNF-α /TNFR2 signaling in MSCs can lead to an increase in their therapeutic efficiency. MSCs isolated from genetically engineered mice lacking TNFR2 had less therapeutic ability than MSCs isolated from normal mice [271, 272].

In a study conducted in 2020 by Ghada Beldi et al., co-culture of WT or TNFR2 KO-MSCs with mouse T cells was used to investigate the role of TNFR2 in the immunomodulatory function of MSCs [273]. Then, T cells were stimulated by antibodies against CD3 and CD28, and by immunostaining, the cytokines related to the stimulated T cell population were evaluated. Also, different Treg markers were examined to check the percentage of these cells in each test group. The results showed the importance of the presence of TNFR2 on the immunomodulatory properties of MSCs. However, the absence of TNFR2 does not eliminate the immunosuppressive potential of MSCs [273]. It was also shown that the production of pro-inflammatory cytokines dependent on TNFR2 was associated with a significant decrease in the groups co-cultured with WT-MSCs [273].

Regarding the status and percentage of Treg cells in different experimental groups, the percentage of T cells induced to Treg was higher in the groups co-cultured with WT-MSCs, and it showed that MSCs expressing TNFR2 have higher Foxp3^+^ Treg induction capacity [273, 274]. Therefore, MSCs can reduce the proliferation, activation, and production of inflammatory cytokines in T cells by expressing TNFR2 [273]. Consequently, it is imperative to investigate the mechanisms related to weakened immunomodulatory in TNFR2 KO-MSCs. In another study to investigate this issue, it was reported that knocking out TNFR2 expression, in addition to decreasing the immunomodulatory ability of MSCs, leads to a decrease in specific markers characterizing these cells (except CD44) [275]. Also, the expression of inflammatory cytokines such as TNF-α, IL-6, and IFN-γ increases significantly in TNFR2 KO-MSCs [275]. This is while the expression of anti-inflammatory cytokines such as IL-10 and TGFβ decreases [275]. The comparison of nitric oxide production by two types of WT-MSCs and TNFR2 KO-MSCs also indicates that nitric oxide production in WT-MSCs expressing TNFR2 is higher than TNFR2 KO-MSCs [275]. Also, knocking out TNFR2 as an immune checkpoint on MSCs can decrease the ability of these cells to migrate and heal wounds [275].

## Concluding and future perspectives

One of the main features that lead to the therapeutic applications of MSCs is their immunomodulatory potential. One of the most recent suggestions for this function of MSCs is the immune checkpoint-related mechanisms. As mentioned in this article, MSCs express different types of ICPs and their ligands. Previously, it was mentioned in many studies that pretreated MSCs in inflammatory conditions, such as adding TNF-α and IFN-γ to the culture medium, can increase their therapeutic potential by improving their immunomodulatory ability (Table 4). The exact mechanism of this issue was not clear. However, in this review, we conclude that in addition to the effect of these pro-inflammatory environments on the production of anti-inflammatory cytokines and exosomes by MSCs, inflammatory conditions by affecting different signaling pathways lead to increased ICPs and their ligands expression on the surface of MSCs and their secretion and thereby increase these cells immunomodulatory potential.

**Table 4:** Strategies for increasing MSCs immunomodulatory potential and their impact on ICPs and ICPLs expression

Modification strategy	Method	Advantage & disadvantage	Effect on MSCs ICPs and ICPls experssion	Examples	Other effects	Ref
Preconditioning	Small molecule and pharmacological priming	Advantage 1. Inexpensive and simple methodology 2. Wide availability of GMP compounds Disadvantage 1. Possible off-target effects on MSC 2. Risk of mutagenesis	Not investigated	Desferrioxamine	1. ↓ Mitochondrial activity 2. Apoptosis of MSCs	[276]
				Dimethyloxalylglycine	1. ↑ HIF-1α, VEGF production 2. ↑ MSC survival	[277]
				Anesthetic isofurane	1. ↑ SDF-1, HIF-1α, and CXCR4 production 2. MSC survival	[278]
				All-trans retinoic acid	1. ↑ COX-2, VEGF, HIF-1α, CCR2, CXCR4, and Ang-2 production 2. ↑ Rat wound healing 3. ↑ IL-6 secretion 4. ↓ Th17 differentiation 5. ↓ TNF-α and IFN-γ production	[279, 280]
				Rapamycin	1. ↑ COX-2 and PGE2 2. ↓ IFN-γ induced MHC-II on MSCs	[281]
			Inhibit Gal-9 secretion from MSCs	α-lactose	↑ T cells proliferation and differentiation to Th1 and Th17 compared to intact MSCs	[228]
	Cytokine priming	Advantage ↑ Immunomodulatory potential Disadvantage 1. Heterogeneity within batches due to culturing and isolation methods 2. Large-scale production safety	↑ PD-L1 expression synergistically in combination with IFN-γ	Pretreat with TNF-α	1. ↓ IL-1β, IL-18 and IL-6 from Kupffer cells in coculture system 2. ↓ AST and ALT in transplantation for liver disease 3. Inhibits the activation of NLRP3 in macrophage	[180, 282]
			↑ ICOSL expression	Pretreat with IL-1β	1. ↑ COX-2, IL-6 and IL-8 expression 2. ↑ CXCR4 expression and migration ability 3. ↑ Secretion of G-CSF	[283, 284]
			Not investigated	Stimulate with IL-6	1. ↑ miR-455-3p in MSC-exosomes 2. ↑ PI3K signaling pathway mediated inhibition macrophage activation	[285]
			1. ↑ PD-L1 experssion 2.↑ Secretion of sEV-PD-L1 from MSCs 3. ↑ Production of soluble Gal-9	Pretreat with IFN-γ	1. ↑ Anti-inflammatory macrophage (M2) differentiation 2. ↑ Treg differentiation 3. ↑ MSC-EVs immunomodulatory effects	[286]
	TLR ligands priming	N/A	1. ↑ PD-L1 experssion 2. ↑ Gal9 expression	Pretreat with Poly I:C (TLR3)	Suppresses T cell proliferation and functions	[227]
			↑ Gal9 expression	1. Pretreat with zymosan (TLR2) 2. Pretreat with LPS (TLR4)	Suppresses T cell proliferation and functions	[227]
Hypoxia priming	1. Using a dedicated hypoxia station 2. Hypoxia chambers 3. Using cobalt chloride (CoCl2)	Advantage ↑ Immunomodulatory and survival Disadvantage 1. Enhances cell proliferation and efficacy during manufacture 2. Requires specific manufacturing equipment	↑ Expression of sCTLA-4	CoCl2	1. ↑ MSCs undifferentiated states duration 2. ↑ MSCs Proliferation and survival 3. ↑ MSC mobilization and homing 4. ↑ HGF, HIF1α, VEGF, IL-6, IL-10, and IDO production from MSCs 5. ↓ Senescence associated β-galactosidase in MSCs	[287]
Genetic engineering	Knockout or knockin 1. Lentivirus 2. Adenovirus 3. Retrovirus 4. Plasmid transfection 5. Zinc finger nuclease (ZFN) 6. TALEN 7. CRISPR/Cas9	Advantage 1. Relative high efficiency 2. Precise gene edition 3. Precise gene edition for CRISPR/Cas9 Disadvantage 1. Off-target effects risk 2. Complicated design 3. Risk of insertional mutagenesis	Variable based on manipulated gene	SOX2, PAX6, OTX2, AGO2	Multiple genes can be targeted for inducible knockout	[35, 288]
				IDO	Affects immune cell proliferation	[289]
				SLCO1A2, SLCO1B3	↓ Cell death in iPSC-derived cardiomyocytes	[290]
				PD-L1	1. ↓ MSCs-sEVs potential to suppress T-cell responses	[191]
	Knockdown 1. siRNA 2. miRNA	N/A	↓ PD-L1 experssion by MSCs	Anti-PD-L1 siRNA	↓ Inducing Treg differentiation	[184]
3D cultures	1. Hanging Drops 2. Non-cross-linked hyaluronic acid gel 3. Multiwell hydrogel system 4. Chitosan films 5. Spheroid dishes 6. Rocker system 7. 3D rotational culture system 8. Ultra-low attachment plates	Advantage 1. ↑ MSC immunophenotypic and molecular profile stability 2. ↑ Angiogenic properties 3. ↑ Exosome production 4. ↑ Cell-to-cell communication 5. ↑ Anti-apoptotic and anti-fibrotic Disadvantage 1. High-cost requirement 2. Heterogonous distribution of cells 3. Size variability depends on the technique	Not investigated	Spheroids	1. ↑ Regenerative and therapeutic effect by suppressing inflammatory responses 2. ↑ IFN-γ, IL-6, FGF2 and HGF experssion 3. ↓ CXCL2/MIP2, TNF-α, IL-1β, and PGE2 expression 4. ↑ Proliferation of MSCs 5. ↑ Trafficking efficacy 6. ↓ Neutrophil activity	[32, 291]

Each of the ICPs and their ligands expressed by MSCs plays an important role in their immunomodulatory ability. A very important point that came to the opinion of the authors during the review of various articles is that in studies that investigated the role of different immune checkpoints expressed by MSCs, the suppression of the pathway related to the studied ICPs led to the loss of MSCs' therapeutic potential. This is while other mechanisms associated with the therapeutic potential of MSCs, such as the production of exosomes, cytokines, growth factors, and mitochondrial transfer, are not affected by the inhibition of the ICPs and their ligands' expression or function. Also, inhibiting the expression or function of one type of ICP and its ligands on the surface of MSCs does not affect other ICPs and their ligands' functions.

In addition, it has been shown in some studies that inhibiting the functions of PD-1 and its ligand leads to a compensatory increase in the expression of proteins related to other ICPs and suppresses the inhibition induced by the treatment. It seems that in some studies, the inhibition of the immunomodulatory function of MSCs by the inhibition of an ICP has been exaggerated to some extent. As discussed in this article, it has been pointed out in some other studies that the inhibition of an ICP and its ligands can lead to a decrease in the therapeutic potential of MSCs. Still, it does not lead to its complete inhibition.

Some studies have pointed out the importance of cancer-associated MSCs (CA-MSCs) in tumor progression using various mechanisms such as immune modulation, increased metastasis, drug resistance, angiogenesis, and, finally, increased tumor growth. However, none of these studies have investigated the importance of the ICPs and their ligands' role expressed or secreted by CA-MSCs. In many tumor treatments using ICBs, only the changed responses in tumor cells and immune cells are examined, and the importance of MSCs in this type of treatment is ignored. It seems that examining the functions of CA-MSCs after treatment with ICBs can help our understanding of the effect of this type of treatment on the interaction between CA-MSCs and tumor cells.

Considering the importance of the expression of ICPs and their ligands by MSCs, various methods, including CRISPR/CAS technology, can be used to produce MSCs with high expression levels of one or more types of ICPs and ligands. Also, considering the role of 3D culture in increasing the therapeutic potential of MSCs, it seems that they affect the expression of ICPs and ligands. Therefore, studies can be designed to investigate this issue. Studies on ICPs and their ligands expressed on the surface of MSCs are low, and it seems that this field is in its early stages, and more studies are needed to reveal all its aspects.

## Data Availability

No datasets were generated or analysed during the current study.

## Abbreviations

MSCs: Mesenchymal stromal/stem cells
EVs: Extracellular vesicles
MSCs-EVs: MSCs-derived EVs
MSCs-CM: MSCs-derived condition medium
MVs: Microvesicles
EXOs: Exosomes
sEVs: Small extracellular vesicles
ICPs: Immune checkpoints
ICPLs: Immune checkpoints ligands
ICBs: Immune checkpoint blockades
HIV: Human immunodeficiency virus
NF-κB: Nuclear factor-kappa B
TNF-α: Tumour necrosis factor-α
Th: T helper cell
Treg: Regulatory T
IL: Interleukin
LPS: Lipopolysaccharides
MDSC: Myeloid-derived suppressor cells
CTLA-4: Cytotoxic T-lymphocyte associated protein 4
PBMCs: Peripheral blood mononuclear cells
NK cells: Natural killer cells
PD-1: Programmed cell death-1
PD-L: Programmed cell death-ligand
IFN-γ: Interferon-gamma
TLR: Toll-like receptor
polyI:C: Polyinosinic-polycytidylic acid
ICOSL: Inducible costimulator ligand
ILC2: Type 2 innate lymphoid cells
ADORA2A: A2A receptor
TGF: Transforming growth factor
IDO: Indoleamine 2,3-dioxygenase
Arg-1: Arginase-1
Gal-9: Galectin-9
TIM3: T cell immunoglobulin and mucin domain-containing protein 3
MAPK: Mitogen-activated protein kinase
TIGIT: T cell immunoreceptor with immunoglobulin and ITIM domain
MDS: Myelodysplastic syndrome
MM: Multiple myeloma
HVEM: Herpes virus entry mediator
BTLA: B and T lymphocyte attenuator
ITIM: Immunoreceptor tyrosine-based inhibitory motif
SHP2: Src homology region 2 domain-containing phosphatase-2
LRMSCs: Lung tissue-resident MSCs
ICC: Intrahepatic cholangiocarcinoma
AMPK: AMP-activated protein kinase
TNFR2: Tumor necrosis factor receptor 2
WT-MSCs: Wild type MSCs
NO: Nitric oxide
